# Successful endoscopic treatment of multiple large intrahepatic bile duct stones with benign choledochojejunal anastomotic stenosis

**DOI:** 10.1055/a-2120-1694

**Published:** 2023-07-13

**Authors:** Akihiko Kida, Takatoshi Yoshio, Hironori Minami, Jun Asai, Kaheita Kakinoki, Takeshi Urabe, Taro Yamashita

**Affiliations:** 1 Department of Gastroenterology, Public Central Hospital of Matto Ishikawa, Hakusan, Japan; 2 Department of Gastroenterology, Kanazawa University Hospital, Kanazawa, Japan; 3 Department of Surgery, Public Central Hospital of Matto Ishikawa, Hakusan, Japan

A 64-year-old man who had undergone subtotal stomach-preserving pancreaticoduodenectomy with modified Child’s reconstruction for ampullary carcinoma presented with fever and jaundice. Blood tests showed cholangitis, and magnetic resonance imaging revealed multiple intrahepatic bile duct stones (IBDSs) ( Fig. 1 ). Therefore, endoscopic treatment was planned. A forward-viewing endoscope (PCF-H290TI; Olympus Medical Systems, Tokyo, Japan) was inserted. The choledochojejunal anastomotic site had a pinhole-like opening ( Fig. 2 ). Endoscopic retrograde cholangiography (ERC) revealed multiple large IBDSs ( Fig. 3 , Fig. 4 ). Based on these findings, multiple large IBDSs with benign choledochojejunal anastomotic stenosis were diagnosed. The strategy of endoscopic treatment was to first dilate the anastomotic site with the placement of a temporary fully-covered self-expandable metal stent (FCSEMS) and remove the stones. The choledochojejunal anastomotic site was dilated by a balloon dilator followed by the placement of two plastic stents and then FCSEMS ( Fig. 5 ). These stents were removed endoscopically after 2 months, and stricture resolution of the choledochojejunal anastomotic site was achieved. IBDSs were observed by peroral direct cholangioscopy (PDCS) using a forward-viewing endoscope (SIF-H290S; Olympus Medical Systems). Electrohydraulic lithotripsy (EHL) for the right IBDSs and crushed stone extraction by a basket under PDCS were performed; however, the left IBDSs remained. One month later, balloon dilatation was performed at the choledochojejunal anastomotic site because it was narrower; similar procedures were performed on the remaining IBDSs, and PDCS and ERC revealed no IBDS residue ( Video 1 ).

**Fig. 1:**
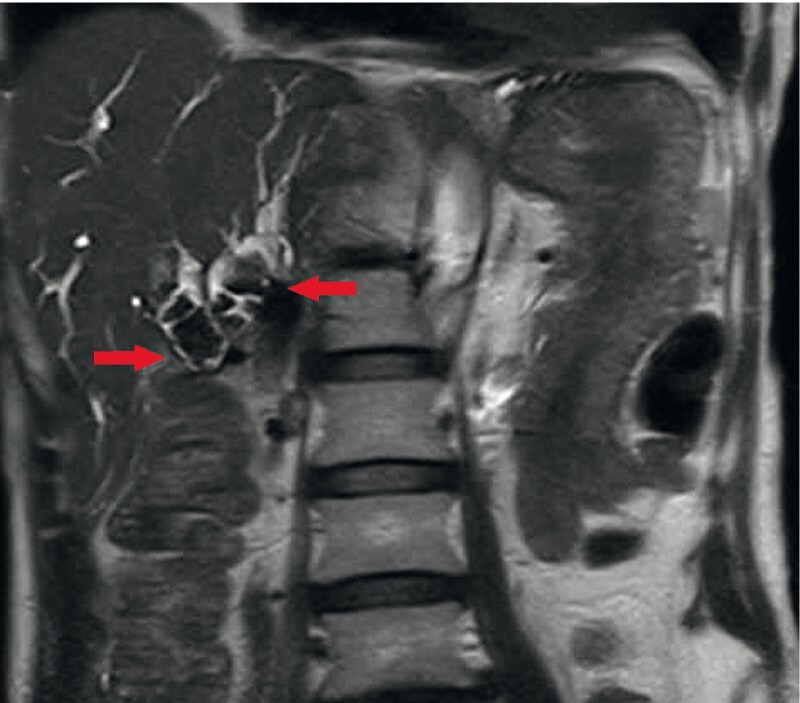
Magnetic resonance imaging revealed dilatation and multiple large stones in the bilateral intrahepatic bile duct. The red arrow shows multiple large bile duct stones.

**Fig. 2:**
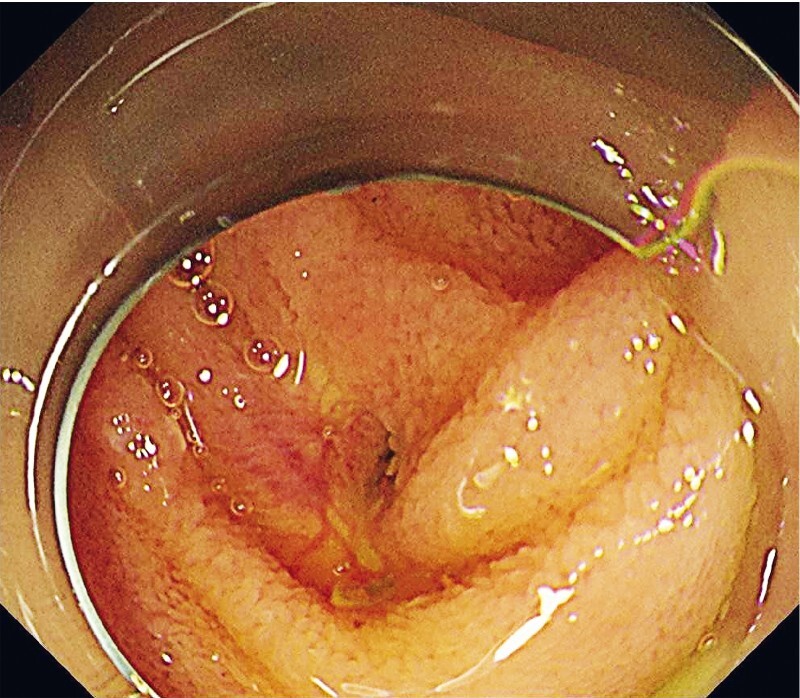
The choledochojejunal anastomotic site had a pinhole-like opening; however, no malignant findings were observed.

**Fig. 3:**
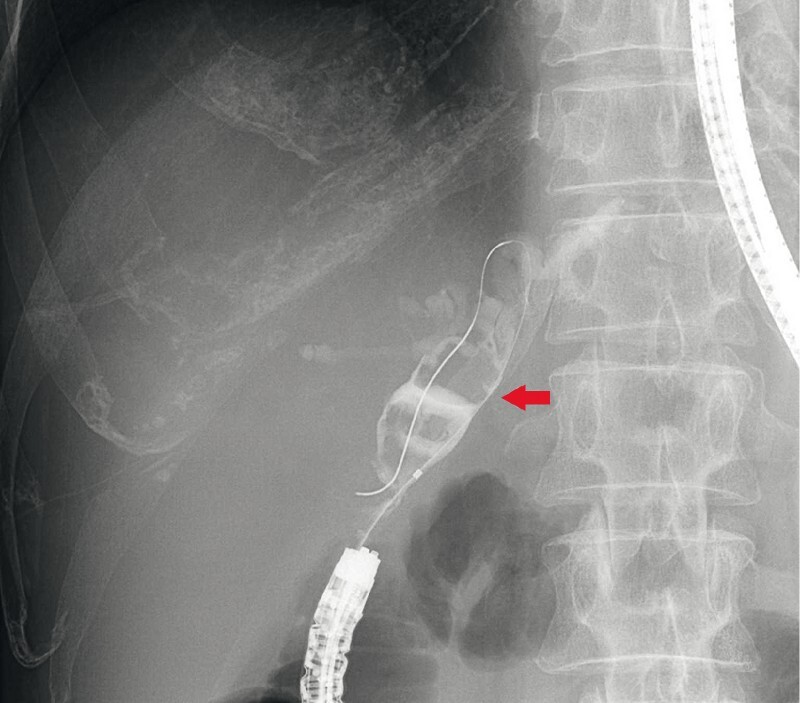
Endoscopic retrograde cholangiography (ERC) of the left intrahepatic bile duct revealed dilatation and multiple large stones with a maximum diameter of 16 mm. The red arrow shows multiple large bile duct stones in the left intrahepatic bile duct.

**Fig. 4:**
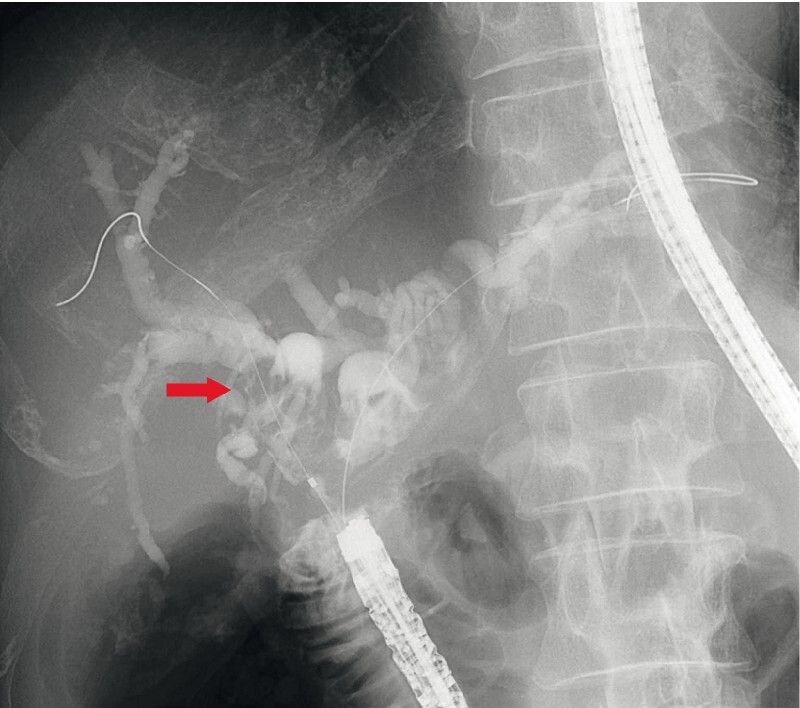
ERC of the right intrahepatic bile duct revealed dilatation and multiple large stones. The red arrow shows multiple large bile duct stones in the right intrahepatic bile duct.

**Fig. 5:**
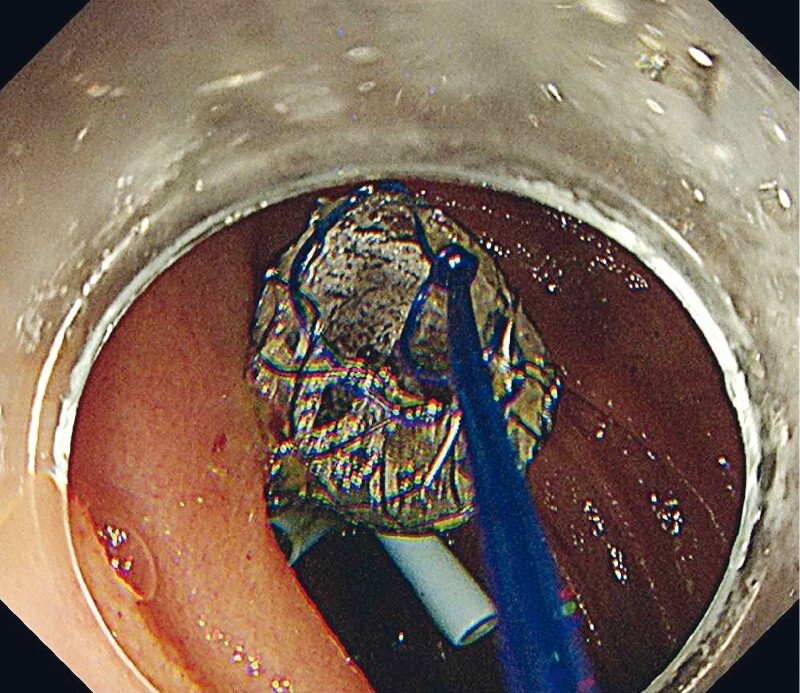
Two plastic stents and a fully-covered self-expandable metal stent were placed at the choledochojejunal anastomotic site.

**Video 1** Successful endoscopic treatment of multiple large intrahepatic bile duct stones with benign choledochojejunal anastomotic stenosis.


The endoscopic treatment of IBDSs with benign choledochojejunal anastomotic stenosis is extremely difficult. The usefulness of placing a FCSEMS for benign biliary strictures including benign choledochojejunal anastomotic stenosis has been demonstrated 1 2 3 . However, few studies have reported the utility of combining FCSEMS placement and EHL under cholangioscopy for bile duct stones with intrahepatic benign biliary strictures and such strictures of the common bile duct as well as the surgically altered anatomy 4 5 . These combined techniques may be useful for complex IBDSs with benign choledochojejunal anastomotic stenosis.

Endoscopy_UCTN_Code_TTT_1AR_2AH

## Notes

Endoscopy E-Videos

https://eref.thieme.de/e-videos
*E-Videos* is an open access online section of the journal *Endoscopy* , reporting on interesting cases and new techniques in gastroenterological endoscopy. All papers include a high-quality video and are published with a Creative Commons CC-BY license. Endoscopy E-Videos qualify for HINARI discounts and waivers and eligibility is automatically checked during the submission process. We grant 100% waivers to articles whose corresponding authors are based in Group A countries and 50% waivers to those who are based in Group B countries as classified by Research4Life (see: https://www.research4life.org/access/eligibility/ ). This section has its own submission website at https://mc.manuscriptcentral.com/e-videos
